# From paradise to precarity: Hawai'i's public health gaps foreshadow U.S. coastal vulnerabilities

**DOI:** 10.1016/j.lana.2025.101259

**Published:** 2025-09-27

**Authors:** Hao Chi, Hua Yang, T. Samuel Shomaker, Youping Deng

**Affiliations:** John A. Burns School of Medicine, University of Hawai'i at Mānoa, Honolulu, Hawai'i, USA

Despite Hawai'i's reputation as an environmentally conscious and globally admired island state, a series of persistent, preventable public health gaps increasingly threaten both health equity and climate resilience. These gaps—ranging from widespread wastewater contamination and extreme physician shortages to a rapidly growing unhoused population—reveal underlying structural vulnerabilities that merit urgent policy attention, not only for Hawai'i but also for coastal jurisdictions across the Americas confronting similar climate and demographic pressures.

Over 83,000 active cesspools in Hawai'i discharge an estimated 50 million gallons of untreated wastewater into the environment daily, contaminating both groundwater and nearshore marine systems. This chronic exposure contributes to increased risks of gastrointestinal illness, ear infections, and skin disease, and regularly triggers beach advisories based on elevated levels of fecal-indicator bacteria. Sea-level rise and extreme rainfall events exacerbate these exposures by elevating groundwater levels and overburdening combined storm-wastewater systems, further facilitating leakage of untreated effluent into public spaces and ecosystems.^1^, ^2^, ^3^, ^4^ Although statewide cesspool conversion is mandated by 2050 under Act 125, progress remains slow in high-risk recharge and coastal zones, particularly in low-income communities lacking financing and technical assistance. Many of the same households face simultaneous climate, health, and economic burdens, amplifying their vulnerability. Structural remediation has lagged behind hydrological risk.^1^

Health workforce fragility compounds these environmental risks. The 2024 Hawai'i physician workforce assessment reports a statewide shortage of 543 full-time equivalents (FTEs), rising to 768 FTEs when adjusted for geographic disparities across islands. Primary care and behavioural health shortages are most severe on the neighbour islands, where access is further constrained by inter-island travel and limited telehealth coverage. These workforce gaps delay the detection and response to communicable diseases such as tuberculosis and pertussis, disrupt vaccination recovery efforts, and increase dependence on costly off-island referrals.^5^

Hawai'i's homelessness rate—approximately 81 per 10,000 residents—is among the highest in the U.S., with 11,637 people counted on a single night in 2024. Congregate shelter settings and limited clinical outreach capacity heighten the risk of tuberculosis transmission and complicate chronic disease management, while mental illness and substance use remain highly prevalent. The concentration of social precarity alongside infectious disease vulnerabilities reflects systemic service fragmentation.^6^, ^7^, ^8^

Compounding these issues are emerging climate-linked health threats. Preliminary surveillance data from the Hawai'i Department of Health suggest 400–600 annual emergency visits for heat-related illness on O'ahu alone. However, Hawai'i currently lacks a binding occupational heat standard. The federal Occupational Safety and Health Administration (OSHA) has proposed but not yet finalised a heat illness prevention rule, while Hawai'i's participation in the National Emphasis Program does not carry enforceable regulatory weight.^9^, ^10^, ^11^

Additional risks stem from wildfires, as intensified by invasive grasses, prolonged drought, and land-use changes. The 2023 Lahaina wildfire underscored the acute respiratory consequences of wildfire smoke, particularly for medically underserved populations. Rising sea levels and groundwater-driven flooding continue to threaten drainage infrastructure and cesspool integrity, further linking climate variability to sanitation-related exposures.^12^

We propose the following urgent policy priorities:

1. Accelerate cesspool conversions with equity-focused financing mechanisms and technical support targeting high-risk recharge zones and low-income households. Without proactive support, timelines will disproportionately fail in the most climate-exposed communities.
2. Realign workforce financing and distribution to account for island geography. This includes expanding graduate medical education (GME) slots, increasing loan repayment incentives for rural placements, scaling community health worker models, and investing in robust telehealth infrastructure.
3. Establish a minimum enforceable climate-health standard, including legally binding protections against heat illness, sustained environmental hazard surveillance (vector-borne disease, wildfire smoke), and modernised public health data systems capable of integrating environmental and social risk factors.

Hawai'i's case should be viewed not as an anomaly, but as a bellwether. Coastal states and territories across the Americas—from the Gulf Coast to the Caribbean—face converging threats of climate disruption, health workforce fragility, and social inequity. By addressing structural gaps in Hawai'i's health and environmental systems now, we can avert wider systemic failures elsewhere.

## Contributors

HC and YD conceived the manuscript. HY and TS contributed clinical and policy contextualisation. HC drafted the original manuscript. YD and TS critically revised the draft. All authors approved the final version. YD was responsible for the decision to submit the manuscript.

## Declaration of interests

The authors declare no competing interests.
